# *LincRNA-BC7* as a Modulator of Olaparib Sensitivity in Triple-Negative Breast Cancer

**DOI:** 10.3390/epigenomes10020034

**Published:** 2026-06-01

**Authors:** Olalekan Olatunde Fadebi, Babatunde Adebola Alabi, Richard Khanyile, Zodwa Dlamini, Rahaba Marima

**Affiliations:** 1 Department of Medical Oncology, Faculty of Health Sciences, Steve Biko Academic Hospital, University of Pretoria, Hatfield, Pretoria 0028, South Africa; u22493591@tuks.co.za (O.O.F.); babatunde.alabi@up.ac.za (B.A.A.); richard.khanyile@up.ac.za (R.K.); 2 SAMRC Precision Oncology Research Unit (PORU), Pan African Cancer Research Institute (PACRI), University of Pretoria, Hatfield, Pretoria 0028, South Africa; zodwa.dlamini@up.ac.za

**Keywords:** breast cancer, *LincRNA-BC7*, *BRCA1*, olaparib, triple-negative breast cancer (TNBC), ceRNA, *miR-663a*, PARP inhibition

## Abstract

Background: Triple-negative breast cancer (TNBC) remains a clinical challenge due to its aggressive nature and the frequent emergence of therapeutic resistance. While the role of protein-coding genes in DNA repair is well-documented, the regulatory contributions of the non-coding genome, specifically long intergenic non-coding RNAs (lincRNAs), remain largely undefined. Objectives: In this study, we characterize the biological significance of *LincRNA-BC7*, a novel transcript identified within the breast cancer field effect. Methods: Through a combined in silico and in vitro approach, we investigated the transcriptional dynamics of the *LincRNA-BC7*/*miR-663a*/*BRCA1* axis in response to the PARP inhibitor, Olaparib. Results: Our results demonstrate that Olaparib induces selective cytotoxicity in *BRCA1*-deficient MDA-MB-231 cells while sparing non-cancerous HEK293 cells, a response accompanied by a significant downregulation of *LincRNA-BC7* and a reciprocal upregulation of *BRCA1*. Bioinformatics analysis through BLASTN, miRBase, and KEGG revealed that *LincRNA-BC7* contains highly complementary binding sites for *miR-663a*, suggesting it functions as a competing endogenous RNA (ceRNA) or “molecular sponge.” Conclusions: By sequestering *miR-663a*, *LincRNA-BC7* appears to modulate the expression of critical signaling nodes within the PI3K-AKT and TP53 pathways, thereby influencing cellular sensitivity to DNA-damaging agents. These findings suggest that *LincRNA-BC7* is a key determinant of the aggressive TNBC phenotype and the response to PARP inhibition. Our study establishes the *LincRNA-BC7*/*miR-663a* axis as a novel biomarker for precision risk stratification and a promising therapeutic target to enhance treatment outcomes in *BRCA1*-associated breast cancers.

## 1. Introduction

### 1.1. Epidemiology of Global and Local Triple-Negative Breast Cancer

Due to its highly aggressive nature, lack of diverse therapeutic alternatives, and historically poor prognosis compared to other malignancies of the breast, triple-negative breast cancer (TNBC) presents a major obstacle in contemporary oncology. Diagnostically, this specific subtype is characterized by the simultaneous absence of estrogen receptors (ER), progesterone receptors (PR), and human epidermal growth factor receptor 2 (HER2), which effectively precludes the use of standard endocrine therapies or HER2-targeted agents that successfully manage other breast malignancies [1]. Because of this unique molecular profile, TNBC remains a primary driver of global cancer-related mortality and morbidity, standing as a critical area of need despite ongoing innovations in modern cancer research.

Globally, breast cancer is the most frequently diagnosed cancer, with approximately 2.3 million new cases and 670,000 deaths recorded annually [2]. TNBC accounts for roughly 10–15% of these cases in Western populations; however, this incidence rises significantly among women of African descent, where it can comprise up to 20–30% of diagnoses in the United States and over 40% in certain West African nations [3].

In the South African context, breast cancer is the leading cancer among women, with a lifetime risk of 1 in 27 [4]. National statistics indicate that black women are disproportionately affected by aggressive phenotypes; local studies have found that TNBC accounts for approximately 17–19% of the breast cancer burden in South African cohorts [5]. Unlike international trends, in which TNBC is strictly linked to younger patients, South African data reveal a complex distribution in which advanced-stage presentation is the dominant pattern across ages, with over 60% of patients diagnosed at Stages III or IV [6]. This is particularly evident in the Gauteng region, where clinical audits at major centers like Steve Biko Academic Hospital and Charlotte Maxeke Johannesburg Academic Hospital show high rates of late-stage disease and high proliferation indices in TNBC cases [4].

### 1.2. Disparities and Risk Factors in Sub-Saharan Africa

Breast cancer is often higher in white women compared to black women; black women often develop an aggressive form of the illness, which often occurs earlier in life [7]. Breast cancer detected in women under age 50 is known as early-onset breast cancer. It is usually aggressive and has a poor prognosis [7]. Compared to women in other LMIC countries, black South African women have also been shown to be more likely to have early-onset breast cancer [8]. Recent studies reported that black women have a higher risk of developing late-stage breast cancer and increased mortality compared to white women [9]. Other risk factors that may contribute to the prevalence of breast cancer in the Sub-Saharan region include heredity, lower fertility rates, obesity, and socio-economic status [10].

### 1.3. Challenges of Identifying Novel Targets in Triple-Negative Breast Cancer

The identification of actionable therapeutic targets in triple-negative breast cancer (TNBC) remains one of the most critical and unyielding bottlenecks in precision oncology [11]. Unlike receptor-positive breast cancer subtypes, which possess well-defined, driver molecular vulnerabilities, such as estrogen receptor (ER) or human epidermal growth factor receptor 2 (HER2) amplification, TNBC is uniquely defined by the lack of targetable surface receptors [12,13]. This absence of traditional, protein-coding driver oncogenes forces a paradigm shift toward unearthing novel candidates within highly intricate, non-coding transcriptomic layers. However, moving away from conventional receptor-centric targets introduces massive biological and analytical hurdles that severely complicate the discovery pipeline.

A primary obstacle in isolating viable targets within this non-coding landscape is the sheer functional complexity of long intergenic non-coding RNAs (lincRNAs) [14]. Unlike classical protein receptors that typically act as simple upstream switches within a linear pathway, these non-coding transcripts operate as multifaceted, multi-layered regulatory networks. They act more like nuanced cellular rheostats that modulate gene expression and fine-tune signaling cascades across multiple pathways simultaneously [15]. Because a single lincRNA candidate can hold regulatory sway over several highly conserved, deeply interconnected survival networks, disrupting them is rarely straightforward [15].

Consequently, attempting to therapeutically silence or manipulate a single novel target frequently triggers immediate biological pushback. The complex architecture of these non-coding networks allows cancer cells to rapidly initiate unpredictable feedback loops and compensatory signaling shunts [16]. When one pathway is blocked, parallel survival mechanisms are immediately upregulated, effectively masking the cell’s true vulnerability and promoting rapid drug adaptation or intrinsic resistance [17]. Furthermore, verifying which candidates are genuine oncogenic drivers rather than passive “passenger” transcripts remains an immense challenge [18]. Transitioning these candidates from basic in silico genomic profiling to reliable functional validation requires navigating a heavily distorted cellular landscape, where isolating a single, stable target that can be therapeutic without causing immediate resistance or widespread cellular compensation remains exceptionally difficult [17,19].

### 1.4. The Emerging Role of Non-Coding RNAs in Breast Cancer Pathogenesis

Breast cancer is a heterogeneous and complex disease characterized by aberrant expression of numerous genes, including inactivation of tumor suppressors and activation of oncogenes. Although about 80% of the human genome is transcribed, only 2% of that transcribed genome codes for proteins [20]. It was believed that only protein-coding genes played a major role in the onset and spread of cancer [19]. However, recent studies have implicated non-coding RNAs as one of the molecular players of breast cancer [21]. Among non-coding RNAs, long intergenic non-coding RNAs (lincRNAs) originate from DNA sequences between two protein-coding genes and have been reported to exert either oncogenic or tumor-suppressive roles in the pathogenesis of breast cancer [22]. Long intergenic non-coding RNAs have become important modulators of cellular signaling networks and gene expression and have been shown to play an increasingly important role in cancer biology in recent studies, especially in the control of gene expression and signaling cascades [23].

### 1.5. Characterization of the LincRNA-BC7/miR-663a Axis

Ding and Zhu investigated the expression of long intergenic non-coding RNAs in breast cancer by comparing tumor tissues with adjacent normal tissues using RNA sequencing [24]. They identified 538 long intergenic non-coding RNAs, of which 124 were exclusively expressed in adjacent normal tissues and 62 in cancer tissues. Additionally, 134 lincRNAs were upregulated and 272 downregulated in breast cancer compared to normal tissue. The study validated the differential expression of four long intergenic non-coding RNAs (BC2, BC4, BC5, and BC8) using PCR. However, the signaling pattern of *LincRNA-BC7*, one of the identified long intergenic non-coding RNAs, in breast cancer remains uncharacterized.

We propose a mechanistic model wherein *LincRNA-BC7* functions as a pivotal regulator of the tumor microenvironment by acting as a competitive endogenous RNA, or “molecular sponge,” for *miR-663a*. Through precise sequence complementarity, *LincRNA-BC7* sequesters *miR-663a*, effectively shielding its downstream targets within the PI3K-AKT and TP53 signaling pathways, which are fundamental to the aggressive phenotype of triple-negative breast cancer (TNBC). Furthermore, this study demonstrates that the *LincRNA-BC7*/*miR-663a* axis modulates *BRCA1* activity, thereby serving as a critical determinant of cellular sensitivity to the PARP inhibitor Olaparib.

### 1.6. Mechanism and Clinical Evolution of Olaparib

Olaparib (Lynparza), the first-in-class poly (ADP-ribose) PARP inhibitor that functions through the principle of synthetic lethality, specifically targeting cancers with deficiencies in the homologous recombination repair (HRR) pathway, such as those with *BRCA1* or *BRCA2* mutations [25,26]. Its primary mechanism of action involves the inhibition of PARP enzymes responsible for repairing single-strand DNA breaks (SSBs); by blocking this repair and “trapping” PARP enzymes at the site of DNA damage, Olaparib causes the collapse of replication forks and the accumulation of lethal double-strand breaks (DSBs) in HRR-deficient cells [27,28]. Historically, Olaparib’s clinical development reached a major milestone in 2014 with its Food and Drug Administration (FDA) approval for *BRCA*-associated ovarian cancer, followed by a significant expansion in 2018 for germline *BRCA*-mutated, HER2-negative metastatic breast cancer [29]. Clinical outcomes in TNBC have been promising, with the landmark Phase III OlympiAD trial demonstrating that Olaparib monotherapy significantly prolongs progression-free survival (PFS) compared to standard chemotherapy [30]. Current therapeutic protocols are exploring its utility in combination with other agents, such as BKM120, to target the PI3K-AKT axis, or alongside chemotherapy to enhance long-term efficacy and improve immune function in patients with advanced TNBC [28].

### 1.7. Study Rationale

In this study, Olaparib was employed in TNBC cell lines as a critical functional probe to characterize the downstream effects of *LincRNA-BC7*. By evaluating cellular response to Olaparib-induced stress, we can effectively measure how *LincRNA-BC7* expression influences *BRCA1* activity and DNA repair capacity, defining its role as a driver of therapeutic sensitivity in aggressive breast cancer phenotypes.

Elucidating the expression profile and functional consequences of *LincRNA-BC7* not only defines its role as a stromal-derived oncogenic driver but also unveils how molecular shifts in the surrounding “normal” tissue actively govern therapeutic resistance and clinical outcomes in breast cancer.

## 2. Results

### 2.1. Cell Viability in Response to Olaparib

**Figure 1:**
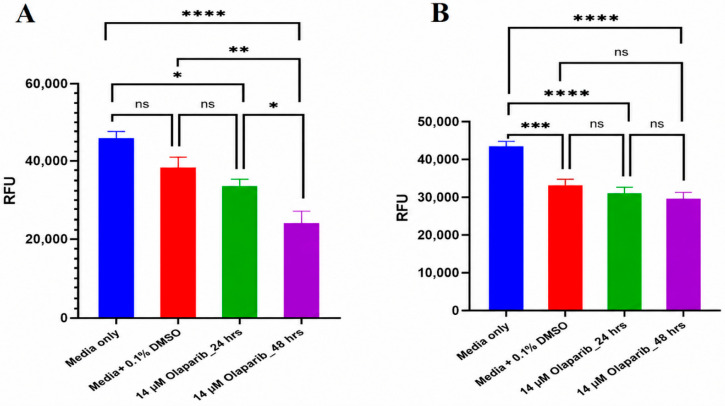
ANOVA and Tukey’s multiple comparison test for (**A**) MDA-MB-231 cells. The 14 μM Olaparib-treated cells show a time-dependent decrease in proliferation compared to the untreated cells. (**B**) The 14 μM Olaparib-treated HEK293 cells show reduced proliferation compared to the untreated cells. Statistical differences were determined with two-way ANOVA followed by Tukey’s post-comparison test. * *p* < 0.05 was considered significant. (not significant, *p* < 0.05), * (*p* < 0.05), ** (*p* < 0.01), *** (*p* < 0.001), and **** (*p* < 0.0001).

The Alamar blue assay was used to assess the viability and proliferation of MDA-MB-231 and HEK293 cells before and after exposure to Olaparib. The assay relies on the physiological reduction of Alamar blue dye, which was measured fluorometrically at 590 nm and 645 nm at 24- and 48 h time points. Figure 1A,B shows the extent of Alamar blue reduction in MDA-MB-231 and HEK293 cells, respectively, following Olaparib treatment.

Figure 1 illustrates that 14 μM Olaparib significantly decreased cell viability and inhibited proliferation in MDA-MB-231 cells over 24 to 48 h.

In contrast, HEK293 cells treated with the same concentration showed no significant changes in viability or proliferation, indicating no anti-proliferative effect (*p* < 0.05).

### 2.2. Gene Expression Analysis of BRCA1 and LincRNA-BC7

**Figure 2:**
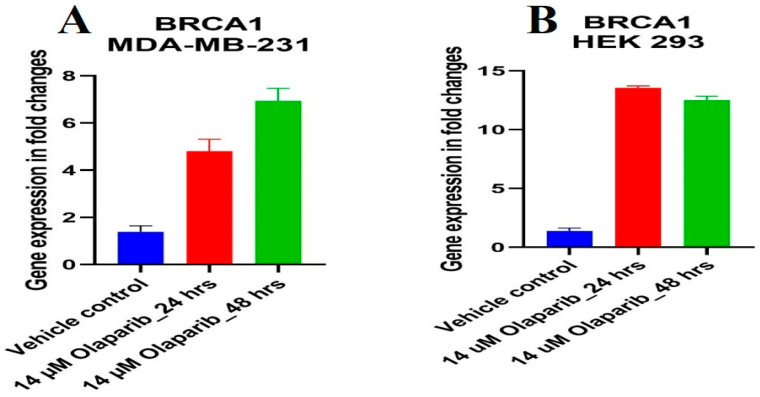
Bar graph representing BRCA1 gene expression levels presented in fold changes at 24- and 48 h time points in MDA-MB-231 and HEK293 cell lines. (**A**) In the MDA-MB-231 cell line, BRCA1 was upregulated. (**B**) In HEK293 cells, BRCA1 was also upregulated.

*BRCA1* gene expression levels in MDA-MB-231 cells were assessed following 24- and 48 h treatments with 14 µM Olaparib. The results revealed a significant upregulation in *BRCA1* expression, indicating a cellular response to Olaparib-induced DNA damage. Similarly, *BRCA1* levels were significantly upregulated in treated HEK293 cells (Figure 2).

However, the expression level of *LincRNA-BC7* in MDA-MB-231 cells treated with 14 µM Olaparib was also evaluated at 24 and 48 h, with results expressed as fold changes (Figure 2). At the 24 h time point, a marked decrease in *LincRNA-BC7* was observed, indicating that Olaparib suppresses its expression in these TNBC cells. However, there was an insignificant upregulation at the 48 h time point.

In contrast, HEK293 cells exposed to the same Olaparib concentration showed a slight, statistically insignificant increase in *LincRNA-BC7* expression at 24 h, followed by a minor, insignificant decrease at 48 h.

**Figure 3:**
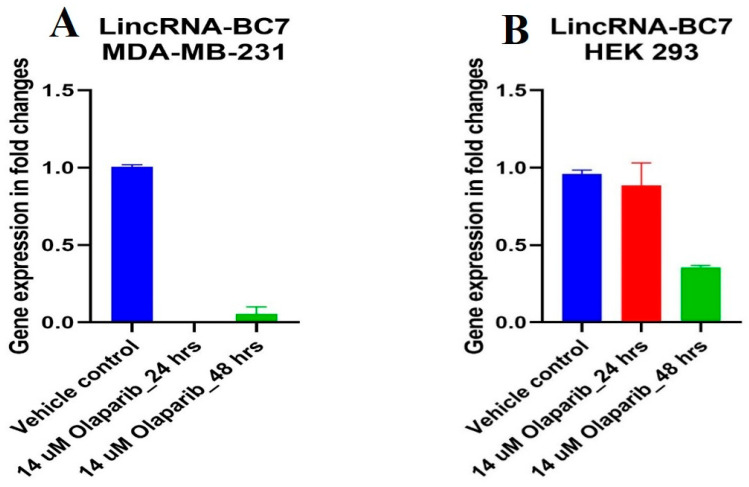
Bar graph representing *LincRNA-BC7* gene expression in fold changes in 24- and 48 h treated (**A**) MDA-MB-231 and (**B**) HEK293 cells.

*BRCA1* and *LincRNA-BC7* expression fold changes are shown in Figure 2 and Figure 3, respectively. The *y*-axis in the graph reflects fold change relative to the untreated control. In the treated MDA-MB-231 cells shown in Figure 3, *LincRNA-BC7* downregulation was consistent across both time points, suggesting that the suppression is not time-dependent. These findings confirm that Olaparib effectively reduces *LincRNA-BC7* expression in *BRCA1*-deficient TNBC cells. A statistical comparison of 24- and 48 h treatments in MDA-MB-231 cells revealed highly significant differences (*p* < 0.05 and *p* < 0.0001).

### 2.3. In Silico Sequence Alignment and Transcriptome Analysis

To evaluate sequence-specific interactions, an initial alignment was performed with BLASTN 2.8.0+ using default parameters. The search targeted the NCBI Transcript Reference Sequences database (*Homo sapiens*, taxid: 9606), specifically querying for complementarity with *miR-663a* (Accession: NR_030386.1). The alignment identified 14 initial hits, which were subsequently filtered for sequence integrity. Two high-confidence hits demonstrating 100% identity over the alignment window were retained for further analysis. The corresponding transcript sequences were retrieved from the NCBI database and aligned against the query (Query_479259) to characterize potential binding motifs.

#### 2.3.1. Expression of *miR-663a* Across Different Tissues

**Figure 4:**
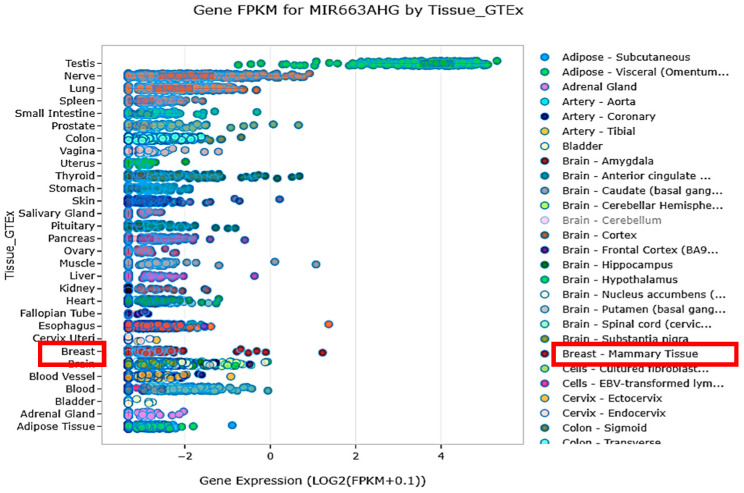
Tissue-specific expression profile of MIR663AHG. RNA sequencing expression levels (quantified as LOG_2_ (FPKM+0.1) are shown across diverse human tissues from the Genotype-Tissue Expression (GTEx) database. Each dot represents an individual tissue sample. The Breast tissue category (in the red boxes) exhibits a distinct clustering pattern, with low-to-moderate MIR663AHG transcript levels relative to high-expressing tissues such as the Testis. FPKM: Fragments Per Kilobase of transcript per Million mapped reads.

Bulk tissue expression analysis using the Genotype-Tissue Expression (GTEx) database (dbGaP Accession phs000424.v.10.p2) revealed that *miR-663a* is most highly expressed in lymphatic nodes. In the context of mammary biology, establishing the expression profile in healthy “Breast-Mammary Tissue” provides a critical baseline for elucidating dysregulated signaling patterns in breast cancer. This reference point is essential as multiple microRNAs may compete for the same binding sites on *LincRNA-BC7*, influencing the overall regulatory landscape of the tumor microenvironment (Figure 4).

#### 2.3.2. Characterization of *LincRNA-BC7* and *miR-663a*

**Figure 5:**
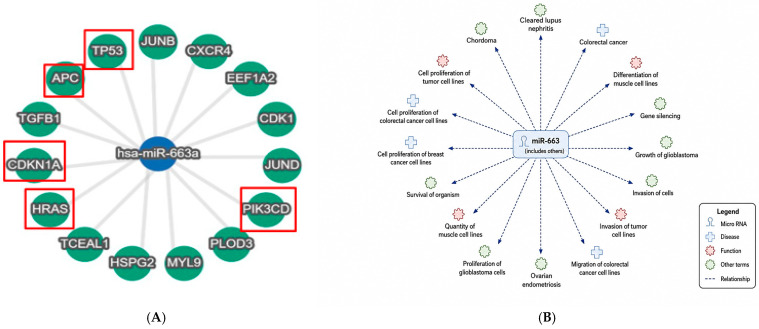
Qiagen IPA (**A**). The interaction network of hsa-miR-663a with several targeted genes indicates a putative regulatory interaction, suggesting that hsa-miR-663a may have regulatory roles affecting multiple signaling pathways and gene expression, potentially impacting processes related to BCa development (In red boxes). Targeted genes indicated in red are implicated explicitly in BCa. (**B**) The influence of miR663A in biological and pathological processes, such as BCa.

Analysis via miRBase (http://www.mirbase.org, accessed 15 October 2024) confirmed a significant match between *LincRNA-BC7* and *miR-663a*, highlighting complementary regions in which the lincRNA may act as a competing endogenous RNA (ceRNA). By sequestering *miR-663a*, *LincRNA-BC7* potentially prevents the microRNA from binding to its experimentally validated mRNA targets, which include APC, CDKN1A, GRB2, HRAS, PIK3CD, and TP53. Integration of Ingenuity Pathway Analysis (IPA) and Gene Ontology (GO) further suggests that this axis is a central regulator of canonical pathways involved in breast cancer pathogenesis (Figure 5).

#### 2.3.3. Potential Pathways Associated with *miR-663a*

**Figure 6:**
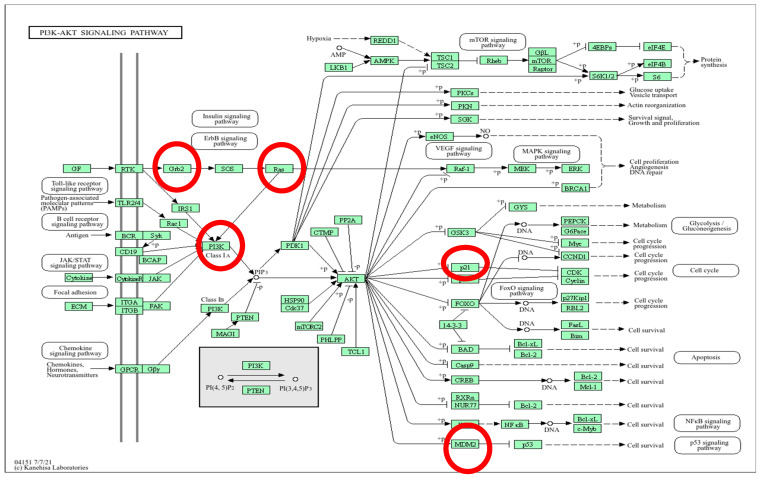
Schematic representation of the PI3K-AKT signaling cascade regulated by the *miR-663a* axis. Functional mapping via the KEGG database identifies *miR-663a* targets, including Ras, PI3K, AKT, p53, and p21 (CDKN1A) (in red cycles), which serve as critical mediators of cellular survival and cell cycle control.

Functional mapping through the KEGG pathway database identified the PI3K-AKT signaling cascade as a primary network influenced by the *miR-663a* axis. Specifically, *miR-663a* targets HRAS, TP53, CDKN1A, and PIK3CD, all of which are critical mediators of cellular proliferation, survival, and growth. In BRCA1-deficient environments, the loss of *miR-663a*-mediated regulation may lead to hyperactivation of PI3K-AKT signaling, thereby promoting aggressive tumor phenotypes and therapeutic resistance (Figure 6).

## 3. Discussion

The spatial localization of *LincRNA-BC7* corresponds to a short ~370 nt region on chromosome 21 (Chr21: 9,825,439–9,825,808; hg19) within tumor-adjacent tissues, offering a compelling vantage point for interrogating the broader breast cancer landscape, especially given its largely uncharacterized signaling architecture. By prioritizing this lincRNA, the present study transcends traditional tumor-intrinsic markers to explore how the peritumoral milieu orchestrates disease progression through the lens of field cancerization.

The experimental results of this study provide critical empirical evidence for the regulatory significance of *LincRNA-BC7* in TNBC. By validating the endogenous expression of this novel transcript in MDA-MB-231 and HEK293 cell lines using strand-specific RT-qPCR and melting curve analysis, we have confirmed that *LincRNA-BC7* is a transcriptionally active entity within the peritumoral environment. This validation is a significant outcome, as it elevates *LincRNA-BC7* from a computationally predicted sequence to a biologically relevant molecule that may drive oncogenic signaling in aggressive breast cancer subtypes. The detection of this lincRNA in both cancerous and non-cancerous models underscores its systemic relevance in the breast cancer field effect, suggesting that its influence extends beyond the immediate tumor mass into the surrounding “normal” tissue.

Our qPCR data following Olaparib treatment revealed a striking, coordinated transcriptional response, clarifying the role of *LincRNA-BC7* in therapeutic sensitivity. In MDA-MB-231 cells, Olaparib exposure triggered a marked and consistent downregulation of *LincRNA-BC7* at both 24- and 48 h intervals. This suppression coincided with a significant reciprocal upregulation of *BRCA1* mRNA, indicating a major shift in the cellular DNA damage response. The inverse relationship between these two molecules supports our proposed mechanistic framework in which *LincRNA-BC7* serves as an oncogenic driver that normally suppresses or interferes with *BRCA1*-mediated repair. By reducing *LincRNA-BC7* levels, Olaparib treatment effectively dismantles this inhibitory axis, attempting to restore *BRCA1* activity even in a deficient genomic background.

The functional consequence of this transcriptional shift was further evidenced by the Alamar Blue cell viability assays, which demonstrated a selective and potent anti-proliferative effect in MDA-MB-231 cells. The significant decrease in viability in these cells, compared to the negligible impact on HEK293 cells, validates the principle of synthetic lethality. While both cell lines showed an upregulation of *BRCA1* in response to drug-induced DNA damage, only the cancerous MDA-MB-231 cells, characterized by baseline *BRCA1* deficiency, succumbed to the cumulative genomic stress. This suggests that, while the cell attempts compensatory upregulation of *BRCA1*, the concurrent suppression of *LincRNA-BC7* may reach a threshold incompatible with survival in aggressive TNBC phenotypes.

The in silico findings of this study provide a robust computational foundation that illuminates the complex regulatory network of *LincRNA-BC7* and its role in TNBC pathogenesis. Using high-stringency BLASTN 2.8.0+ alignments against the NCBI Transcript Reference Sequences database, we identified a 100% identity region between the novel transcript and *miR-663a*. This sequence-level precision is vital, as it transitions the study from broad transcriptomic observations to a defined, sequence-specific interaction model. The discovery of these high-confidence binding motifs provides structural evidence for the proposal that *LincRNA-BC7* operates as a functional competing endogenous RNA (ceRNA), specifically evolved to interact with the *miR-663a* seed region.

The integration of GTEx tissue-specific expression data adds a significant layer of biological context to these sequence alignments. The observation that *miR-663a* is highly expressed in lymphatic nodes and maintains a stable baseline in healthy mammary tissue suggests that its dysregulation in the tumor microenvironment is a departure from a tightly controlled physiological state. In the aggressive landscape of TNBC, *LincRNA-BC7* acts as a “molecular sponge,” disrupting homeostasis by sequestering *miR-663a* and preventing it from carrying out its natural tumor-suppressive functions. This computational model effectively explains how a previously uncharacterized lincRNA can orchestrate broad signaling shifts by modulating the availability of a single microRNA hub.

Furthermore, functional mapping through KEGG, IPA, and TargetScan extends the impact of this axis to critical downstream effectors of cell survival. The in silico prediction that the *LincRNA-BC7*/*miR-663a* axis regulates targets such as CDKN1A (p21), PIK3CD, and TP53 suggests that this network is a master regulator of the PI3K-AKT signaling cascade. In *BRCA1*-deficient environments, the loss of microRNA-mediated control over these pathways leads to the hyperactivation of proliferation and growth signals, driving therapeutic resistance. These computational insights provide the mechanistic “why” behind the experimental observations, framing *LincRNA-BC7* as a central coordinator of the aggressive TNBC phenotype and a high-impact target for precision oncology intervention.

## 4. Materials and Methods

### 4.1. Cell Line and Cell Culture

The human TNBC cell line, MDA-MB-231 (Cat No: 50-238-5044) and the human embryonic kidney cell line, HEK293 (ATCC CRL-1573), were purchased from ATCC (Manassas, VA, USA). Dulbecco’s modified Eagle medium (DMEM, Invitrogen, Carlsbad, CA, USA) containing 10% fetal bovine serum and 100 μg/mL penicillin (Invitrogen, Carlsbad, CA, USA) was used to culture the cells mentioned above in 5% CO_2_ and 95% humidity at 37 °C. Cells were always maintained in the logarithmic growth phase, trypsinized with 0.25% trypsin-EDTA, and subcultured into freshly prepared media every 72 h. MDA-MB-231 and HEK293 cells (1 × 10^5^ cells/well) were seeded into 96-well plates and allowed to adhere overnight.

### 4.2. Drug Treatment and Cell Viability Assay

Olaparib (MedChemExpress, Monmouth Junction, NJ, USA) was prepared as a 100 mM stock solution in DMSO, aliquoted, and stored at −20 °C until use. The concentration value of the working solution of Olaparib used was 14 µM. After 24 h of cell starvation, cells were exposed to either blank growth medium (control) or growth medium containing 14 µM concentrations of Olaparib (treatment medium) for 24 and 48 h at 37 °C and 5% CO_2_. The final DMSO concentration in all treatment conditions was maintained at 0.1% to minimize solvent-related toxicity. Following overnight incubation, 10 μL of Alamar Blue reagent (Bio-Rad, Hercules, CA, USA) was added to each well containing 100 μL of culture medium. The excitation fluorescence wavelength was set to 590–645 nm (Ex/Em), and emission was detected at 590 nm using the Gen5TM microplate reader (Synergy HT, Biotech, Winooski, VT, USA). Readings were taken alternatively from the top and bottom of each well.

The selection of 14 μM Olaparib was based on preliminary dose–response characterization across TNBC cell lines, representing a calculated “window of opportunity” dose. This concentration allows for the observation of the modulatory role of *LincRNA-BC7* without inducing massive, non-specific cell death that would saturate the DNA repair machinery and obscure subtle molecular interactions [28]. Furthermore, this dose aligns with the peak plasma concentrations (Cmax) observed in clinical pharmacokinetic profiles, ensuring that the in vitro observations reflect therapeutic exposures achievable in patients.

The selection of the Alamar Blue (resazurin) assay as the primary endpoint for assessing cell viability in response to Olaparib was based on its superior sensitivity and non-destructive nature compared to traditional assays such as 3-(4,5-dimethylthiazol-2-yl)-2,5-diphenyltetrazolium bromide (MTT) or Sulforhodamine B (SRB). Unlike the MTT assay, which requires cell lysis and the dissolution of insoluble formazan crystals, limiting it to a single, terminal snapshot, Alamar Blue is a non-toxic, water-soluble redox indicator [31]. This allows continuous monitoring of the same cell populations over time, which is essential for studying the cumulative effects of Olaparib-induced PARP trapping and the subsequent progression of double-strand breaks [26]. Furthermore, while SRB measures total cellular protein as a proxy for cell density, Alamar Blue specifically monitors the metabolic activity of living cells by measuring the reduction of resazurin to the highly fluorescent resorufin by mitochondrial and cytoplasmic enzymes [32]. This distinction is critical in TNBC research, as Olaparib treatment can induce senescence or delayed apoptosis, in which cell mass may remain constant while metabolic viability declines significantly. By using a fluorometric approach, we achieved a higher dynamic range and sensitivity, enabling the accurate quantification of subtle variations in viability mediated by the *LincRNA-BC7*/*miR-663a* axis, free of the confounding effects of reagent-induced toxicity.

### 4.3. Gene Expression

RNA was extracted from MDA-MB-231 and HEK293 cells using a Qiagen RNeasy extraction kit (Hilden, Germany). Subsequently, 0.5 μg of total RNA was reverse transcribed using the qMax cDNA Synthesis Kit (Accuris Instruments, Edison, NJ, USA) according to the manufacturer’s instructions. The reaction mix used is detailed in Table 1.

Complementary DNA (cDNA) was amplified using the qMax Green qPCR Master Mix (Accuris Instruments, Edison, NJ, USA) with gene-specific primers on a real-time PCR system. PCR amplification was performed using a thermal cycler, PikoReal^®^ real-time PCR, version 2.2 (Thermo Fisher Scientific, Waltham, MA, USA), according to the cycling conditions outlined in Table 2, following the manufacturer’s protocol.

The specific primers used in this study were designed using the NCBI Primer-BLAST version 2.5.0 tool as follows: *LincRNA-BC7*, 5′-CCTTCCGGCGTCCCA-3′ (Forward) and 5′-GGCCACCAGGAAAACACG-3′ (Reverse); *BRCA1*, 5′-GCTGATCCCCTGTGTGAGAG-3′ (Forward) and 5′GGCATTTGATTCAGACTCCCC-3′ (Reverse); β-actin, 5′-CGACAGGATGCAGAAGGAGAT-3′ (Forward) and 5′-CAAGAAAGGGTGTAACGCAACTA-3′ (Reverse).

### 4.4. In Silico Bioinformatics Analysis

To investigate sequence-specific interactions, an initial alignment was performed using BLASTN version 2.8.0+ (https://www.ncbi.nlm.nih.gov, accessed 12 October 2024) with default parameters. The analysis targeted the NCBI Transcript Reference Sequences database (*Homo sapiens*) to identify regions of complementarity with *miR-663a* (Accession: NR_030386.1). Out of 14 initial hits, two high-confidence transcripts demonstrating 100% identity over the alignment window were selected for detailed characterization of potential binding motifs. Downstream target genes for the identified miRNA were predicted using KEGG (https://www.kegg.jp/, accessed 13 October 2024) and TargetScan (http://www.targetscan.org/vert_72/, accessed 13 October 2024), and functional enrichment analysis for TNBC was performed using DAVID Bioinformatics Resources, version 2024q4 (https://davidbioinformatics.nih.gov/, accessed 14 October 2024).

### 4.5. Statistical Analysis

All statistical analyses were performed using GraphPad Prism version 10.4.1 (GraphPad Software, San Diego, CA, USA). Data are presented as means ± standard error of the mean (SEM) derived from at least three independent biological replicates, each performed in triplicate. The normality of the data distribution was first verified to ensure the appropriateness of parametric testing.

Statistical significance was determined using a two-way analysis of variance (ANOVA), which was selected to simultaneously evaluate the influence of two independent variables, treatment condition and time, and to identify any synergistic interactions between them. To control for the family-wise error rate associated with multiple comparisons, Tukey’s post hoc test was subsequently applied; this provided a rigorous framework for identifying specific significant differences between individual treatment groups while minimizing Type I error (false positives). A probability (*p*) value < 0.05 was considered statistically significant, with further significance levels denoted as (*p* < 0.01), *** (*p* < 0.001), and **** (*p* < 0.0001).

## 5. Conclusions

This study identifies *LincRNA-BC7* as a critical molecular determinant of therapeutic response in TNBC. Our findings demonstrate that *LincRNA-BC7* acts as a ceRNA, sequestering *miR-663a* to modulate the PI3K-AKT signaling pathway and thereby influencing cellular sensitivity to the PARP inhibitor Olaparib.

The therapeutic management of TNBC remains one of the most formidable challenges in clinical oncology due to its inherent heterogeneity and the lack of traditional hormonal targets. This study focused on the functional characterization of *LincRNA-BC7* and its influence on the efficacy of the PARP inhibitor, Olaparib. Our findings elucidate a sophisticated regulatory hierarchy, the *LincRNA-BC7*/*miR-663a*/*BRCA1* axis, which appears to serve as a critical rheostat for synthetic lethality and therapeutic response in aggressive breast cancer cells. The cornerstone of this study was the observation of selective cytotoxicity in MDA-MB-231 cells compared to non-cancerous HEK293 cells. This differential response validates the principle of synthetic lethality, a concept pioneered in the context of PARP inhibitors [33,34]. By inhibiting PARP-mediated base excision repair in a background of compromised *BRCA1* function, we successfully induced “genomic catastrophe” in the cancer model. Crucially, our data shows that Olaparib treatment induces a coordinated downregulation of *LincRNA-BC7* and a concomitant upregulation of *BRCA1*. This inverse relationship is pivotal; as established by recent studies, the degree of BRCAness or the functional deficiency of homologous recombination is the primary determinant of PARP inhibitor sensitivity [33]. Our study suggests that *LincRNA-BC7* acts as a “molecular brake” on *BRCA1* expression, suggesting that Olaparib’s downregulation of *LincRNA-BC7* may represent a compensatory or regulatory feedback loop attempting to restore genomic stability.

The most significant mechanistic insight from our in silico analysis (IPA, KEGG, and miRBase) is the proposal of *LincRNA-BC7* as a competitive endogenous RNA (ceRNA) or “molecular sponge.” This model aligns with the landmark “ceRNA hypothesis” proposed by Salmena et al. (2011), which suggests that non-coding RNAs communicate through shared microRNA response elements [35]. By sequestering *miR-663a*, *LincRNA-BC7* effectively prevents the microRNA from binding to and degrading *BRCA1* transcripts. When Olaparib suppresses *LincRNA-BC7*, the liberated *miR-663a* should, in theory, target its downstream effectors. However, we observed an upregulation of *BRCA1*, suggesting a more complex regulatory interplay potentially involving the PI3K-AKT and TP53 pathways. As noted by Poliseno et al. (2010) in their work on the *PTEN* pseudogene, disruption of these sponge networks can lead to massive shifts in oncogenic signaling, as we observed here by modulating pathways associated with tumor progression and therapeutic escape [36].

A striking finding of this research is the localization of *LincRNA-BC7* within the peritumoral milieu. This supports the theory of “Field Cancerization,” a concept first introduced by Slaughter et al. (1953) and later refined by Dakubo et al. (2015) [37,38]. Our results suggest that the surrounding “normal” tissue is not a passive bystander but is molecularly primed with *LincRNA-BC7* signatures that may orchestrate disease outcomes. If the peritumoral environment mirrors the epigenetic and transcriptional state of the tumor, it suggests that the “molecular field” could govern drug sensitivity and contribute to the high rates of recurrence seen in TNBC. This observation aligns with more recent work that demonstrated that lncRNAs in the tumor microenvironment can modulate systemic drug responses [39].

Despite these findings, several limitations must be acknowledged. This study primarily provides a predictive framework based on in vitro and in silico methodologies. Future research should also utilize in vivo cancer models, such as mouse xenografts or patient-derived xenografts (PDX), to validate these findings. While the present study elucidates a novel molecular mechanism by which the *LincRNA-BC7*/*miR-663a*/*PI3K-AKT* axis modulates Olaparib sensitivity, the transition to animal models is essential to account for the complex physiological interactions within the tumor microenvironment that cannot be fully replicated in cell culture [26]. This study focused on a clinically relevant concentration of 14 μM to explore the *LincRNA-BC7*/*miR-663a* axis; we recognize that a full dose–response characterization is essential. Future work will include expanded IC50 and Selectivity Index (SI) determinations across a broader panel of non-cancerous and TNBC cell lines to further quantify the precision of this synthetic lethal interaction.

Furthermore, to transition from correlation to causation, future studies must employ direct molecular perturbations [40]. We propose using lentiviral vectors to stably overexpress *LincRNA-BC7* to confirm if increasing this sponge successfully sequesters *miR-663a* and decreases Olaparib sensitivity. Conversely, implementing CRISPR-Cas13 or siRNA-mediated knockdown of *LincRNA-BC7* will test whether its depletion restores *miR-663a* activity and enhances the efficacy of PARP inhibition.

This study contributes to the growing body of research highlighting the role of lincRNAs in shaping the “BRCAness” phenotype and the therapeutic vulnerability of aggressive breast cancer subtypes. By elucidating the *LincRNA-BC7*/*miR-663a*/*PI3K-AKT* axis, we provide a molecular rationale for why certain TNBC patients may exhibit differential responses to PARP inhibition despite similar clinical profiles. From a clinical perspective, these findings suggest that *LincRNA-BC7* could serve as a predictive biomarker to refine patient selection for Olaparib treatment, potentially moving TNBC management toward a more personalized, stratified approach. Furthermore, targeting this axis may offer a synergistic strategy to overcome Olaparib resistance, a major hurdle in current oncological protocols.

## Figures and Tables

**Table 1:** cDNA Synthesis Reaction setup (AccurisTM, Denver, CO, USA).

Component	20 µL Reaction	Final Concentration
5× cDNA Synthesis reaction buffer	4 µL	1×
20× qMax Reverse Transcriptase	1 µL	1× added before total RNA
Total RNA, 0.5 µg	Variable	Added up to 0.5 µg
RNAse-free H_2_O	VFinal = 20 µL	

**Table 2:** The thermal cycling conditions.

Component	Temperature	Time	Number of Cycles
Pre-amplification	95 °C	5 min	1
Amplification	95 °C	10 s	45
	60 °C	30 s	
	72 °C	30 s	
Cooling	40 °C	30 s	

## Data Availability

The raw data used in this study will be made available from the corresponding author upon reasonable request.

## Abbreviations

ANOVA	Analysis of variance
BLASTN	Basic Local Alignment Search Tool for Nucleotides
*BRCA1*	Breast cancer gene 1
*BRCA2*	Breast cancer gene 2
cDNA	Complementary DNA
ceRNA	Competing endogenous RNA
DAVID	Database for Annotation, Visualization and Integrated Discovery
DMEM	Dulbecco’s modified Eagle medium
DMSO	Dimethyl sulfoxide
DSBs	Double-strand breaks
DSI/NRF	Department of Science and Innovation/National Research Foundation
FDA	Food and Drug Administration
FPKM	Fragments Per Kilobase of transcript per Million mapped reads
GO	Gene Ontology
GTEx	Genotype-Tissue Expression
HRR	Homologous recombination repair
IPA	Ingenuity Pathway Analysis
KEGG	Kyoto Encyclopedia of Genes and Genomes
lincRNAs	Long intergenic non-coding RNAs
MTT	3-(4,5-dimethylthiazol-2-yl)-2,5-diphenyltetrazolium bromide
PACRI	Pan African Cancer Research Institute
PARP	Poly (ADP-ribose) polymerase
PDX	Patient-derived xenografts
PFS	Progression-free survival
POCP	Precision Oncology and Cancer Prevention
PORU	Precision Oncology Research Unit
qPCR	Quantitative polymerase chain reaction
SAMRC	South African Medical Research Council
SARChI	South African Research Chairs Initiative
SEM	Standard error of the mean
SRB	Sulforhodamine B
SSBs	Single-strand DNA breaks
TNBC	Triple-negative breast cancer
